# LAPAROSCOPIC ABDOMINOPERINEAL RESECTION WITH SACRECTOMY: TECHNICAL DETAILS AND PITFALLS

**DOI:** 10.1590/0102-6720201700040016

**Published:** 2017

**Authors:** Haroldo Jose Siqueira IGREJA-JUNIOR, Vilson Leite BATISTA, Bruno dos Santos Viana CARVALHO, Lucas Simões TAVARES, Juliana Gonçalves COELHO

**Affiliations:** 1 Service of General and Oncological Surgery, Portuguese Society of Charity of Campos, Campos dos Goytacazes, RJ, Brazil

**Keywords:** Rectal neoplasms, Rectum, Colorectal surgery, Sacrum, Laparoscopy, Neoplasias retais, Reto, Cirurgia colorretal, Sacro, Laparoscopia

## INTRODUCTION

A total of 16,660 new cases of colon and rectum cancer in men and 17,620 in women are estimated for 2016 in Brazil^2^. In locally advanced rectum cancer, survival after R0 resection is very good, and exenteration should be offered to patients with advanced primary or recurrent tumor, where resection is necessary in addition to total excision of the conventional mesorectum^6^. In the case of invasion of the sacrum, excision with free margins greatly increases the morbidity and radicality of the procedure, posing a challenge to the surgeon.

To date, the highest level of evidence for the benefits of the laparoscopic approach in rectal cancer comes from the Corean Trial^5^ and NCCN^6^ studies. However, the literature lacks data to justify the use of laparoscopy in locally advanced tumors. In Brazil, there is no report of abdominoperineal resection associated with videolaparoscopic sacrectomy.

The purpose of this report is to present an alternative for the treatment of malignant rectal cancer with posterior invasion involving a combined anterior laparoscopic approach and subsequent tumor resection.

## TECHNIQUE

**FIGURE 1:**
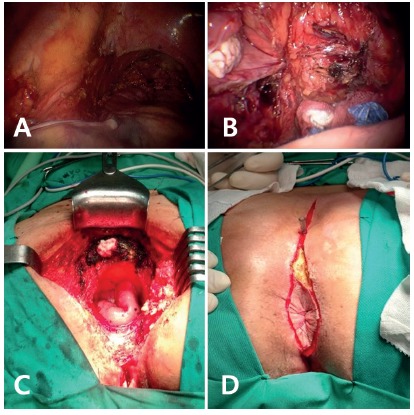
Abdominoperineal resection technique associated with videolaparoscopic sacrectomy: A) mesorectum dissection: point for initial dissection; B) colectomy marking point: using cut dressing to do it; C) perineal time with vision after dissection to sacroiliac joint; D) perineal incision and posterior flap

It begins with the placement of four trocars, two in the upper and the lower right quadrant, one in the umbilical region and one in the left iliac fossa. Unlike the usual, where the dissection of the posterior aspect of the mesorectum would begin, in the described case there was invasion of the sacrum by the tumor. It was decided, then, to begin the dissection by its left lateral aspect, with dieresis of the medial insertion of the left mesocolon, followed by the lateral wing of the rectum, dissection of the Told line, and of the Trietz fascia, progressing to the endopelvic fascia, to the point of tumoral fixation to the sacrum (Figure 1A). After lymphadenectomy of the trunk of the inferior mesenteric artery with preservation of its trunk and the left colic artery, was incised the left mesocolon until the point of the colectomy. The tumor fixation point was demarcated using a cutaway dressing, introduced by the 12 mm trocar for posterior transrectal identification (Figure 1B). There was also left in-bloc salpingo-oophorectomy and the creation of a terminal colostomy in the left iliac fossa.

At this moment the change of decubitus was made to the switchblade position, prone position, for the beginning of perineal time. After perineal incision (Fig. 1C) and fasciomiocutaneous gluteal flap preparation, the dieresis of the sacrococcygeal insertion of the pelvic diaphragm and the rectovaginal septum was performed, followed by the endopelvic fascia to the anterior dissection area, and the lateral sacral ligaments, to sacroiliac joint bilaterally (Figure 1D). A posterior laminectomy was performed at the S3-S4 level, with dissection and reduction of the dural sac at the end of the equine tail. The sacral osteotomy was followed at the same level, using a pneumatic saw and resection with hemostasis by bipolar sealing of the presacral fascia and the lower and middle sacral branches, ending the procto-sacralectomy, with extraction of the surgical specimen. The sacral foramina of the vertebrobasilar plexus were obliterated by titanium screw bilaterally and sealing of the medullary foramen by fibrin sealant, as well as the sacral bloody area. The inferior narrow of the pelvis was obliterated by bladder retroversion, together with fixation of the Proceed^®^ double-sided mesh. It was ended with v-y synthesis of perineal flap and perineal cavitary drainage. Bleeding estimated at the end of the operation was 200 ml.

The patient had a good evolution in the postoperative period, staying the first day in intensive care unit, and the others in infirmary. Motor physical therapy was started on the second day, removing her from the bed. She was discharged seven days afterwards, wandering without help. There were no complications.

## DISCUSSION

The laparoscopic approach in rectal cancer should follow principles: the surgeon’s experience in performing total video excision of the mesorectum, not being indicated in locally advanced tumors and contraindicated in obstructive tumors^6^. Despite this, some authors have reported success in the laparoscopic approach of locally advanced disease. Several factors contribute to the success of the procedure: the team’s experience in advanced videosurgery, availability of materials, interdisciplinary discussion and thorough preoperative planning.

The level of local involvement of the sacrum is a determinant of radicality and surgical morbidity. In cases of coccyx and S5 invasion, sacral resection is performed without major problems. However, if there is a more proximal involvement, it is more complex and can evolve with uncontrollable hemorrhage, neurological injury and urinary complications^7^. In the case described, the invasion was at the S3 level and the resection did not evolve with complications. In T4 tumors, recurrent tumors or small positive margins, the alternative is to expose the surgical site to intraoperative radiotherapy¹.

Bleeding during sacrectomy is critical to the operation. The pre-sacral venous plexus and basal-vertebral sacral veins are the most closely related. Several tamponade methods have been suggested. Topical hemostatic agents, such as fibrin sealants, are options for controlling blood loss. Serious cases that cannot be controlled quickly can result in high mortality. In this case, after sectioning of the sacrum, the tool “Super Jaw” was used for hemostasis and section of the pre-sacral venous plexus and also were used absorbable hemostats. 

This approach proved to be adequate and with little blood loss. No blood products were administered.
